# Current situation and issues regarding termination of risk management plans in Japan

**DOI:** 10.3389/fmed.2024.1387652

**Published:** 2024-05-30

**Authors:** Natsuko Kameyama, Aoi Hosaka, Hideki Maeda

**Affiliations:** ^1^Department of Regulatory Science, Graduate School of Pharmaceutical Science, Meiji Pharmaceutical University, Tokyo, Japan; ^2^Department of Regulatory Science, Faculty of Pharmacy, Meiji Pharmaceutical University, Tokyo, Japan

**Keywords:** RMP termination, risk management plan, drug safety, pharmacovigilance, reexamination system, safety concerns, Japanese drug regulatory system

## Abstract

**Introduction:**

In Japan, drugs approved after the 2013 implementation of the risk management plan (RMP) have the opportunity to be evaluated for RMP termination. However, the guidelines for risk management following the termination of an RMP remain unclear. Drugs are evaluated for RMP termination at the timing of reexamination. Reexamination system is unique to Japan and initiated in 1979, verifies the approved efficacy and safety of a newly marketed drug based on the data from its actual use over a certain period. This study investigated drugs in Japan for which the RMP requirement was lifted upon reexamination and those for which it was not. We organized their characteristics and considered future issues.

**Methods:**

We identified drugs with RMPs and obtained information on RMP termination from the public website of the Pharmaceuticals and Medical Devices Agency (PMDA). The survey period spanned 10 years, from April 2013, when the RMP was implemented, to March 2023.

**Results:**

During the survey period, 72 drugs with RMPs completed reexamination in Japan. The RMP requirement was lifted for 69 drugs (95.8%) and remained for three drugs (4.2%). Upon RMP termination, 15 out of 69 drugs (21.7%) had important potential risks not listed in the package insert, with malignant neoplasm being the most common. Eleven drugs (15.9%) had important missing information not listed in the package insert, with the most common being the impact on cardiovascular risk. Two drugs (2.9%) had ongoing additional pharmacovigilance activities, and 43 drugs (62.3%) had additional risk minimization activities.

**Conclusion:**

Upon reexamination completion, the RMP requirement was lifted for many drugs and remained for a few. Should safety concerns require continued attention following reexamination, we advocate for the continuation of the RMP, guided by more explicit rules. In light of the harmonization of RMP rules with those of other countries, there is a desire for enhanced drug safety management.

## 1 Introduction

A risk management plan (RMP) is a document that outlines the systematic approach to managing drug safety from the development phase through the post-marketing phase. It is designed to ensure the safety of drugs and is mandated to be established at the time of approval (1–3). Since 2013, the formulation and implementation of an RMP have been prerequisites for drug approval in Japan. The requirement for an RMP is lifted when it is confirmed that there are no issues. These issues include whether: (1) The risks and risk minimization materials listed in the RMP have been adequately disseminated in the medical setting. (2) There are no new safety concerns. (3) There has been no significant change in the occurrence of existing risks. (4) Missing information has been collected. Even after the requirement for an RMP is lifted, standard safety measures such as issuing necessary warnings and collecting safety information continue (4).

Drugs are evaluated for RMP termination at the timing of reexamination. The reexamination system is unique to Japan and initiated in 1979, verifies the approved efficacy and safety of a newly marketed drug based on the data from its actual use over a certain period. The outcome of the reexamination can lead to continuation of the drug’s current approval, modification or deletion of the indication, or cancellation of the approval (5–7). Drugs approved post-2013, when the RMP was implemented, are also being reexamined sequentially after a designated reexamination period. Concurrently, these drugs are evaluated for potential RMP termination. Despite the possibility of RMP termination, clear guidelines for risk management post-RMP termination are lacking (8). According to a survey conducted among companies, responses varied after the requirement for an RMP was lifted. These variations included whether to continue the RMP as an internal document, whether to continue risk minimization activities, and whether to change signal evaluation criteria (9). To our knowledge, no other studies have investigated the termination of RMP.

In this study, we investigated drugs for which the requirement for an RMP was lifted (RMP-terminated drugs) and drugs for which it was not lifted (RMP-continuing drugs). We organized their characteristics and considered future issues.

## 2 Materials and methods

In this study, we identified drugs with RMPs from the public information provided by the Pharmaceuticals and Medical Devices Agency (PMDA). We designated drugs as “RMP-continuing drugs” if the requirement for an RMP had not been lifted and the reexamination for all indications had been completed. We also checked the PMDA website’s “List of drugs for which the formulation and implementation of RMP as a condition for approval has been lifted” (4) and designated all listed items as “RMP-terminated drugs.” The survey period spanned 10 years, from April 2013, when the RMP was implemented, to March 2023. We investigated “RMP-terminated drugs” and “RMP-continuing drugs” to determine whether the safety concerns (important identified/potential risks, important missing information) listed in the RMP submitted at the time of reexamination are included in the current package inserts, whether additional pharmacovigilance activities are ongoing, and whether there are additional risk minimization activities. We also investigated the characteristics of “RMP-continuing drugs.”

The RMPs submitted at the time of reexamination and the current package inserts were obtained from the PMDA website’s “Information Search for Prescription Drugs” (10). For “RMP-continuing drugs,” we created a unique database from publicly available information, identified drugs with RMPs approved after April 2013, for which the requirement for an RMP had not been lifted, and for which reexamination for all indications had been completed. This study was prepared in accordance with the strengthening the reporting of observational studies in epidemiology (STROBE) reporting guidelines for cross-sectional studies (11). All statistical analyses were performed using JMP Pro 15, with two-sided p-values less than 0.05 considered statistically significant. The Fisher’s exact test was used for comparisons between categorical data.

## 3 Results

From April 2013 to March 2023, a total of 72 drugs with RMP underwent reexamination for all indications. The background information is detailed in Table 1. When classified by therapeutic indications, the most common were neuropsychiatric and diabetes drugs, each comprising 8 drugs, or 11.1% of the total. Other hormonal drugs (including antihormonal drugs), accounted for 7 drugs, or 9.7%. There were 17 drugs (23.6%) indicated for pediatric use and 1 drug (1.4%) for orphan diseases.

**TABLE 1:** Overview of background information.

		N	%
**Drugs with RMP that have undergone reexamination for all indications**		**72**	
Therapeutic indication classification*	Other hormonal drugs (including antihormonal drugs)	7	9.7
	Antihypertensive drugs	3	4.2
	Antiepileptic drugs	3	4.2
	Neuropsychiatric drugs	8	11.1
	Diabetic drugs	8	11.1
	Follicular/Progestin hormone agents	4	5.6
	Others	41	
Pediatric indication		17	23.6
Orphan drugs		1	1.4

*If there were multiple therapeutic indication classification categories for one drug, each was counted as multiple.

Out of the drugs studied, 69 (95.8%) had terminated their RMPs, while 3 (4.2%) were still continuing with their RMPs. Among the RMP-terminated drugs, none had important identified risks not listed in the package insert after the termination of the RMP. However, 15 drugs (21.7%) had important potential risks, and 11 drugs (15.9%) had important missing information not listed. At the time of RMP termination, 2 drugs (2.9%) had ongoing additional pharmacovigilance activities, and 43 drugs (62.3%) had additional risk minimization activities. For the RMP-continuing drugs, all safety concerns were included in the current package insert. At the time of the survey, none of these drugs had ongoing additional pharmacovigilance activities, but all 3 drugs (100%) had additional risk minimization activities (Table 2).

**TABLE 2:** Status of risk management plan (RMP) termination/continuation and details of safety concerns/additional activities for drugs post-reexamination.

Reexamination completed drugs (*N* = 72)	Number of drugs (%)	Drugs with important identified risks not listed in package inserts	Drugs with important potential risks not listed in package inserts	Drugs with important missing information not listed in package inserts	Drugs with ongoing additional pharmacovigilance activities	Drugs with additional risk minimization activities
RMP-terminated drugs	69(95.8)	0	15(21.7)	11(15.9)	2(2.9)	43(62.3)
RMP-continuing drugs	3(4.2)	0	0	0	0	3(100)

In this study, we examined the safety concerns not listed in the package inserts of 69 drugs that had terminated their RMPs. At the time of reexamination application of these drugs, we identified 164 important potential risks in the last RMPs, of which 23 risks (14.0%) were not included in the package inserts. The most common risks not listed were related to malignant neoplasms (9 risks), accounting for 31.0% of the total number of important potential risks related to malignant neoplasms (29 risks). This was significantly higher than that for other important potential risks (*p* = 0.0073). Seven out of nine of these risks were for drugs indicated for diabetes. Other important potential risks not listed in the package insert were related to infection, the cerebral cardiovascular system (3 risks each), antibody production, the urinary system, administration error (2 risks each), disseminated intravascular coagulation, and suicide-related events (1 risk each) (Table 3). We also found 92 items of important missing information, of which 16 items (17.4%) were not included in the package inserts. The most common missing information not listed was the impact on cardiovascular risk, accounting for 100% of the total number of missing information regarding this issue (seven items). This was significantly higher than other missing information (*p* < 0.0001). All seven items pertained to the drugs indicated for diabetes. They were marked as missing information owing to the lack of information specific to Japanese individuals or regarding long-term administration. Other important missing information not included in the package insert was related to safety when administered to patients with liver dysfunction (5 items), safety when administered to patients with cardiovascular disease, drug interactions, safety during long-term administration, and safety during administration under actual usage conditions (1 item each) (Table 4).

**TABLE 3:** Detailed analysis of important potential risks for the 69 drugs with terminated risk management plans (RMPs).

	Total *N* (risks)	Not listed in package inserts (risks)	%	*p*-value
**Important potential risks**	**164**	**23**	**14.0**	
Malignant neoplasm	29	9	31.0	0.0073
Infectious disease	13	3	23.1	0.3965
Cerebral cardiovascular risks	10	3	30.0	0.1493
Antibody production	5	2	40.0	0.1446
Urinary system risks	7	2	28.6	0.2548
Medication error	6	2	33.3	0.1986
Disseminated intravascular coagulation	1	1	100.0	0.1402
Suicide-related events	6	1	16.7	1.0000

**TABLE 4:** Comprehensive details of important missing information for the 69 drugs with terminated risk management plans (RMPs).

	Total *N* (items)	Not included in the package insert (items)	%	*p*-value
**Important missing information**	**92**	**16**	**17.4**	
Impact on cardiovascular risk	7	7	100.0	< 0.0001
Safety when administered to patients with liver dysfunction	19	5	26.3	0.3083
Safety when administered to patients with cardiovascular disease	1	1	100.0	0.0284
Drug interactions	3	1	33.3	0.4402
Safety during long-term administration	5	1	20.0	1.0000
Safety during administration under actual use conditions	3	1	33.3	0.4402

In our study of the 69 drugs that had terminated their RMPs, we investigated the additional pharmacovigilance activities and risk minimization activities that were ongoing at the time of reexamination application. There were two ongoing additional pharmacovigilance activities: one postmarketing clinical trial (50.0%) and one special drug use-results survey regarding long-term use (50.0%). We investigated details in the respective reexamination reports (12, 13). The post-marketing clinical trial was conducted with the aim of enabling continued administration of the drug to subjects who had obtained a therapeutic effect in the clinical trial, with only one Japanese patient receiving the drug. The special drug use-results survey collected 1,461 cases, surpassing the target number of 1,000 cases. Ongoing additional risk minimization activities included creating and distributing materials for patients (33 activities, 47.8%), creating and distributing materials for healthcare professionals (29 activities, 42.0%), and reporting the occurrence of side effects on company websites (3 activities, 4.3%). Other activities included measures to prevent administration errors, the creation and provision of patient cards, ensuring distinguishability from existing formulations with different concentrations, and safety measures related to pain associated with chronic low back pain and osteoarthritis (one activity each, 1.4%) (Table 5).

**TABLE 5:** In-depth details of ongoing additional activities for the 69 drugs with terminated risk management plans (RMPs).

	Ongoing	Details	Number	%
Additional pharmacovigilance activities	2	Post-marketing clinical trials	1	50.0
		Special drug use-results survey (survey regarding long-term use)	1	50.0
Additional risk minimization activities	69	Creation and distribution of materials for patients	33	47.8
		Creation and distribution of materials for healthcare professionals	29	42.0
		Reporting the occurrence of side effects on company websites	3	4.3
		Measures to prevent administration errors	1	1.4
		Creation and provision of patient cards	1	1.4
		Ensuring distinguishability from existing formulations with different concentrations	1	1.4
		Safety measures for pain associated with chronic low back pain and osteoarthritis	1	1.4

The drugs that were still continuing with their RMPs were Concerta tablets (methylphenidate hydrochloride), Lamictal tablets (lamotrigine), and Botox Vista Injection (botulinum toxin type A) (14–16). The details were investigated in the respective reexamination reports (17–19). Two of these three drugs were neuropsychiatric drugs. Concerta tablets had drug dependence noted in the reexamination report, and the approval conditions were altered after the reexamination, mandating stricter distribution management. Lamictal tablets, an antiepileptic drug, had previously had a drug safety alert (blue letter) (20) issued regarding serious skin disorders. For Botox Vista Injection, distribution management was mentioned as an additional risk minimization activity. Upon checking Botox Injection (21, 22), which has a different RMP from Botox Vista Injection but contains the same ingredients, it was found that reexamination had not yet been completed for all indications.

## 4 Discussion

Upon completion of the reexamination, it was demonstrated that the formulation and implementation of the RMP as a condition for approval were lifted for a significant majority of drugs (95.8%). The three RMP-continuing drugs (4.2%) might share characteristics such as being neuropsychiatric drugs, antiepileptic drugs, having drug dependence, distribution control, and past blue letter issuance. However, RMP-terminated drugs also exhibited these same characteristics (six for neuropsychiatric drugs, two for antiepileptic drugs, four for drug dependence, none for distribution control, and one for past blue letter issues). This suggests that there were no discernible characteristic differences between the RMP-continuing drugs and the RMP-terminated drugs. According to a questionnaire survey, a company voluntarily stated in its reexamination application materials that the RMP of a certain drug, for which a blue letter had been issued, would be continued, and the regulatory authority agreed as proposed (9). However, even for Yaz combination tablets (drospirenone/ethinyl estradiol betadex), for which the RMP was terminated, a blue letter had been issued in the past for thrombosis (23). For one drug, the approval condition was changed at the time of reexamination, and for another drug, reexamination for a drug with the same ingredient had not been completed, suggesting that the continuation of the RMP might depend on timing. However, given the small number of RMP-continuing drugs, it was challenging to identify the characteristics that led to the continuation of the RMP.

Upon the completion of the reexamination process, a significant number of RMPs were terminated. However, we posit that there may be a larger number of RMPs that are worth continuing. Our study revealed that there were safety concerns that were not included in the package insert for drugs that had terminated their RMPs. As for important potential risks, 14.0% of the potential risks were not listed in the package insert at the time of RMP termination. If these potential risks persist after the

reexamination, a challenge arises as there will be no mechanism to confirm them conveniently in the clinical setting post-RMP termination. A study conducted by Saito et al. demonstrated that safety concerns could potentially trigger severe adverse reactions (24). Although the study examined safety concerns in the J-RMP at the time of approval, not reexamination, we believe it is fair to say that at least caution is necessary if safety concerns persist at the time of reexamination. We suggest that potential risks should be included in the package insert if the RMP is terminated, similar to how some drugs mention non-clinical results or risks associated with similar drugs. Otherwise, it would be preferable to continue RMP until every potential risk is either removed or elevated to an identified risk. The purpose of the RMP in Japan is to consolidate all risk management into a single document and ensure through evaluation. We believe that this purpose should remain consistent pre- and post-reexamination. Moreover, among the important potential risks not listed in the package insert upon RMP termination, risks related to malignant neoplasms were the most prevalent. The proportion of important potential risks not listed in the package insert was significantly higher than other potential risks. The reexamination reports for these drugs did not mention the removal of important potential risks. Instead, they stated “No new safety concerns were observed” or “The information is insufficient and the causal relationship with this drug is unclear. Therefore, we will not add any additional information to the package insert and will continue to collect similar information in the future.” However, given that malignant neoplasms require time to develop pathologically, it is often challenging to identify risks during the reexamination period. For instance, six out of nine risks related to malignant neoplasms not listed in the package insert were associated with incretin-related drugs. It took approximately 10 years from initial approval to the completion of reexamination, a period deemed insufficient to assess the risks of the occurrence of malignant neoplasms pathologically. Therefore, in such instances, it is preferable to continue the RMP, rather than terminating it leading only to routine pharmacovigilance activity. Regarding important missing information, 17.4% of the items were not included in the package insert. Among the missing information not listed in the package insert upon RMP termination, missing information regarding the impact on cardiovascular risk was the most common. The proportion of important missing information not listed in the package insert was significantly higher than other missing information. Of the seven items, two specified the deletion of missing information in the reexamination reports, while the remaining five did not include such instructions. Instead, they included statements such as “No new safety concerns were observed” or “We will continue our routine pharmacovigilance activity and will consider whether to issue a warning in the package insert depending on the situation of the event occurrence.” In these cases, the period from initial approval to completion of reexamination was less than 10 years, which is considered to be a pathologically insufficient period to evaluate cardiovascular risk. Consequently, if there is insufficient information regarding risks that take time to manifest or insufficient information regarding safety during long-term administration, it is preferable to continue the RMP. Based on these findings, it is better to decide whether to continue the RMP based on specific criteria, such as continuing the RMP if there are safety concerns that necessitate ongoing attention or terminating the RMP if there are no safety considerations.

Among the conditions currently specified for lifting the RMP formulation and implementation as a condition for approval, there are “no new safety concerns” and “no significant changes in the manifestation of existing risks.” However, there are no standards regarding potential risks that require continued attention, which would be beneficial to add to the rules. Additionally, although it is clearly stated that “information about missing information has been sufficiently collected,” the reality is that RMPs were terminated even if the information was insufficiently collected, necessitating a reconsideration of whether the rules are being appropriately applied.

From a risk assessment and management perspective, periodic safety reports (7, 25, 26) are required during the reexamination period, with report documents being prepared in line with the contents of the RMPs. However, once the reexamination is completed, there ceases to be a regular opportunity for risk assessment, leaving this responsibility to individual companies. According to a previous survey, companies varied in their approach to changing the signal evaluation criteria post-reexamination and in their decision to continue the RMP as an internal document even after the requirement for an RMP as a condition for approval was lifted (9). Furthermore, when the RMPs were terminated, 62.3% of drugs had additional risk minimization activities listed in the RMP, many of which were related to the preparation and distribution of materials to patients and healthcare professionals. The same survey revealed differences among companies in their decision to cease additional risk minimization activities when the RMP was terminated, with three companies stopping and six continuing. There was a general consensus that it was challenging to determine whether to continue these activities, as the government had not provided clear guidance (9). Therefore, it would be beneficial for the government to provide guidelines to replace the RMP regarding risk management after the RMP is terminated. The same survey also highlighted the inconvenience of not being able to view risk minimization materials on the PMDA website after the RMP was removed from the approval conditions at the end of the reexamination period. From this perspective as well, guidance may be needed on effective ways to continue risk minimization activities even after the RMP has been terminated.

In the context of overseas RMPs, there is no mention of termination in the guidelines regarding the RMP of the EU (EU-RMP) (27, 28). The EU-RMP primarily focuses on safety concerns that require special attention and establishes individual pharmacovigilance activity or risk minimization activity for each safety concern. Safety concerns are removed from the EU-RMP if they are determined to be unnecessary (28, 29). As for risk evaluation and mitigation strategies (REMS) in the United States, it is not created for all drugs, but only when it is necessary to implement additional measures beyond the “Precautions for Use,” and when it is not necessary, it will be excluded (30). What they have in common is that they focus only on safety concerns or drugs that require specific action. Conversely, the Japanese RMP (J-RMP) is different because its purpose is to consolidate risk management into one document and ensure that evaluations are surely performed (1). In the J-RMPs, all safety concerns are broadly described. This includes not only those that necessitate special attention but also those for which routine pharmacovigilance activities and risk minimization activities are required (31). However, post-reexamination, the RMP continues only for drugs that require special attention, leading to a discrepancy in the policies pre- and post-reexamination in Japan. The RMP is a dynamic document that undergoes periodic reviews. Actions such as adding important identified risks, reclassifying potential risks to identified risks, incorporating additional risk minimization activities, and deleting missing information are undertaken. Yet, the removal of potential risks or additional risk minimization activities often does not occur until reexamination (9, 32). While utilizing reexamination as an opportunity to review safety concerns is beneficial, a more proactive review of the RMP, such as removing potential risks or additional risk minimization activities before reexamination, is advisable. It would also be beneficial to establish certain rules, like monitoring potential risks and missing information that would take time to evaluate, by continuing the RMP even post-reexamination. Furthermore, despite many pharmaceutical products being sold overseas, regulatory requirements differ between Japan and other countries. This could potentially lead to confusion, given that there are many safety concerns described in the J-RMP but not in the EU-RMP. In the future, there will likely be an increase in cases where the J-RMP is terminated and the EU-RMP continues. Despite the trend of globalization, regulatory RMP requirements vary across jurisdictions worldwide (33, 34). However, aligning them to the same standards would help eliminate confusion. On the other hand, the REMS in the United States differs significantly from J-RMP or EU-RMP, making harmonization difficult and likely to remain an issue in the future.

Our study does have certain limitations. First, the J-RMP system is relatively new, and as a result, there are currently not many subjects available for investigation. In this study, we conducted our research based on the information available 10 years after the implementation of the RMP, but it is crucial to closely monitor future trends as the number of drugs that have an RMP and will undergo reexamination is expected to increase. Second, as there is a time lag until the completion of the reexamination is reflected on the PMDA website, there may be some drugs that are not included in the list of RMP-terminated products, even though the formulation and implementation of the RMP as a condition for approval have been lifted. However, this number is very small and is not considered to significantly impact the results of this study.

In conclusion, upon completion of the reexamination, it was demonstrated that the formulation and implementation of the RMP, as a condition for approval had been lifted for many drugs, with a few exceptions. As risk management becomes more thorough, the importance of safety concerns will change over time. Therefore, we support the termination of the RMP when the conditions are met, utilizing the existing reexamination system as an opportunity to evaluate RMP terminations. However, there may be more RMPs that are better to continue with, and we propose clarifying the criteria for deciding whether to terminate RMPs and provide flexibility for continuing them. It would be beneficial to establish rules and take measures, such as continuing the RMP if there are safety concerns that require ongoing attention. As many pharmaceutical products are expected to undergo reexamination and have their RMPs terminated in the future, we believe that addressing the issues that will arise when the RMP is terminated will lead to recommendations for an appropriate J-RMP system. Better drug safety management is desired considering the unification of RMP rules with other countries.

## Data availability statement

The raw data supporting the conclusions of this article will be made available by the authors, without undue reservation.

## Ethics statement

This study did not require institutional review board approval or patient informed consent because it was based on publicly available information and involved no patient records.

## Authors contribution

NK: Conceptualization, Data curation, Formal analysis, Investigation, Methodology, Writing–original draft, Writing–review and editing. AH: Data curation, Formal analysis, Writing–review and editing. HM: Conceptualization, Funding acquisition, Supervision, Writing–review and editing.
