# Correction: Current situation and issues regarding termination of risk management plans in Japan

**DOI:** 10.3389/fmed.2026.1889775

**Published:** 2026-06-16

**Authors:** Natsuko Kameyama, Aoi Hosaka, Hideki Maeda

**Affiliations:** 1 Department of Regulatory Science, Graduate School of Pharmaceutical Science, Meiji Pharmaceutical University, Tokyo, Japan; 2 Department of Regulatory Science, Faculty of Pharmacy, Meiji Pharmaceutical University, Tokyo, Japan

**Keywords:** RMP termination, risk management plan, drug safety, pharmacovigilance, reexamination system, safety concerns, Japanese drug regulatory system

There was a mistake in Tables 2, 3 as published. In Table 2, the number of drugs with important potential risks not listed in package inserts for RMP-terminated drugs was displayed as “16 (23.2)”. The correct value is “15 (21.7)”. The corrected Table 2 appears below.

**Table 2:** Status of risk management plan (RMP) termination/continuation and details of safety concerns/additional activities for drugs post-reexamination.

Reexamination completed drugs (*N* = 72)	Number of drugs (%)	Drugs with important identified risks not listed in package inserts	Drugs with important potential risks not listed in package inserts	Drugs with important missing information not listed in package inserts	Drugs with ongoing additional pharmaco-vigilance activities	Drugs with additional risk minimization activities
RMP-terminated drugs	69 (95.8)	0	15 (21.7)	11 (15.9)	2 (2.9)	43 (62.3)
RMP-continuing drugs	3 (4.2)	0	0	0	0	3 (100)

**Table 3:** Detailed analysis of important potential risks for the 69 drugs with terminated risk management plans (RMPs).

	Total *N* (risks)	Not listed in package inserts (risks)	%	*p*-value
Important potential risks	164	23	14.0	
Malignant neoplasm	29	9	31.0	0.0073
Infectious disease	13	3	23.1	0.3965
Cerebral cardiovascular risks	10	3	30.0	0.1493
Antibody production	5	2	40.0	0.1446
Urinary system risks	7	2	28.6	0.2548
Medication error	6	2	33.3	0.1986
Disseminated intravascular coagulation	1	1	100.0	0.1402
Suicide-related events	6	1	16.7	1.0000

In Table 3, the total number and percentage of important potential risks not listed in package inserts were displayed as “24” and “14.6”, respectively. The correct values are “23” and “14.0”, respectively. The *p*-values in Table 3 were also recalculated accordingly. The *p*-values for malignant neoplasm, infectious disease, cerebral cardiovascular risks, antibody production, urinary system risks, medication error, disseminated intravascular coagulation, and suicide-related events were corrected from “0.0165”, “0.4083”, “0.1648”, “0.1557”, “0.2721”, “0.2130”, “0.1463”, and “1.0000” to “0.0073”, “0.3965”, “0.1493”, “0.1446”, “0.2548”, “0.1986”, “0.1402”, and “1.0000”, respectively. The corrected Table 3 appears below.

In the abstract, there was an error in the number and percentage of drugs with important potential risks not listed in package inserts in the **Results** Section. The sentence was displayed as:

“Upon RMP termination, 16 out of 69 drugs (23.2%) had important potential risks not listed in the package insert, with malignant neoplasm being the most common.” This has been corrected to read:

“Upon RMP termination, 15 out of 69 drugs (21.7%) had important potential risks not listed in the package insert, with malignant neoplasm being the most common.”

The number and percentage of RMP-terminated drugs with important potential risks not listed in package inserts were incorrect. In the Section **Results**, Paragraph 2, the sentence was displayed as:

“However, 16 drugs (23.2%) had important potential risks, and 11 drugs (15.9%) had important missing information not listed.”

A correction has been made to the Section **Results**, Paragraph 2:

“However, 15 drugs (21.7%) had important potential risks, and 11 drugs (15.9%) had important missing information not listed.”

The number and percentage of important potential risks not listed in package inserts and the *p*-value for malignant neoplasms were incorrect. In the Section **Results**, Paragraph 3, the sentences were displayed as:

“At the time of reexamination application of these drugs, we identified 164 important potential risks in the last RMPs, of which 24 risks (14.6%) were not included in the package inserts.”

“This was significantly higher than that for other important potential risks (*p* = 0.0165).”

Corrections have been made to the Section **Results**, Paragraph 3:

“At the time of reexamination application of these drugs, we identified 164 important potential risks in the last RMPs, of which 23 risks (14.0%) were not included in the package inserts.”

“This was significantly higher than that for other important potential risks (*p* = 0.0073).”

The percentage of important potential risks not listed in package inserts at the time of RMP termination was incorrect. In the Section **Discussion**, Paragraph 2, the sentence was displayed as:

“As for important potential risks, 14.6% of the potential risks were not listed in the package insert at the time of RMP termination.”

A correction has been made to the Section **Discussion**, Paragraph 2:

“As for important potential risks, 14.0% of the potential risks were not listed in the package insert at the time of RMP termination.”

The original version of this article has been updated.

